# Intensity-Modulated Radiotherapy with Regional Hyperthermia for High-Risk Localized Prostate Carcinoma

**DOI:** 10.3390/cancers14020400

**Published:** 2022-01-13

**Authors:** Sota Nakahara, Takayuki Ohguri, Sho Kakinouchi, Hirohide Itamura, Takahiro Morisaki, Subaru Tani, Katuya Yahara, Naohiro Fujimoto

**Affiliations:** 1 Department of Therapeutic Radiology, University Hospital of Occupational and Environmental Health, Kitakyushu 807-8555, Japan; sotanakahara@med.uoeh-uoeh.ac.jp (S.N.); kakino365@gmail.com (S.K.); itamura@med.uoeh-u.ac.jp (H.I.); takam1989@med.uoeh-u.ac.jp (T.M.); s-tani@med.uoeh-u.ac.jp (S.T.); 2 Department of Radiotherapy, Kurashiki Medical Center, Kurashiki 710-8522, Japan; k-yahara@med.uoeh-u.ac.jp; 3 Department of Urology, University of Occupational and Environmental Health, Kitakyushu 807-8555, Japan; n-fuji@med.uoeh-u.ac.jp

**Keywords:** hyperthermia, intensity-modulated radiotherapy, prostate cancer, thermal dose

## Abstract

**Simple Summary:**

Several randomized controlled trials have shown that concurrent use of deep regional hyperthermia and radiotherapy results in a significant increase in local control of cervical and rectal cancer. Intensity-modulated radiotherapy (IMRT) plus androgen deprivation therapy (ADT) has recently become standard treatment for high-risk localized prostate carcinoma; however, as there is room for improvement in outcomes, we have been using hyperthermia to improve the effect of IMRT. This retrospective analysis shows that addition of regional hyperthermia to IMRT plus ADT is a promising approach as it improves clinical outcomes with acceptable toxicity. Importantly, a higher thermal dose was significantly correlated with better biochemical disease-free survival. Further investigations, including prospective trials with detailed treatment protocols, are needed.

**Abstract:**

Background: The purpose of this study was to evaluate the efficacy and toxicity of adding regional hyperthermia to intensity-modulated radiotherapy (IMRT) plus neoadjuvant androgen deprivation therapy (ADT) for high-risk localized prostate carcinoma. Methods: Data from 121 consecutive patients with high-risk prostate carcinoma who were treated with IMRT were retrospectively analyzed. The total planned dose of IMRT was 76 Gy in 38 fractions for all patients; hyperthermia was used in 70 of 121 patients. Intra-rectal temperatures at the prostate level were measured to evaluate thermal dose. Results: Median number of heating sessions was five and the median total thermal dose of CEM43T90 was 7.5 min. Median follow-up duration was 64 months. Addition of hyperthermia to IMRT predicted better clinical relapse-free survival. Higher thermal dose with CEM43T90 (>7 min) predicted improved biochemical disease-free survival. The occurrence of acute and delayed toxicity ≥Grade 2 was not significantly different between patients with or without hyperthermia. Conclusions: IMRT plus regional hyperthermia represents a promising approach with acceptable toxicity for high-risk localized prostate carcinoma. Further studies are needed to verify the efficacy of this combined treatment.

## 1. Introduction

Radiation therapy with androgen deprivation therapy (ADT) is the main treatment modality for patients with high-risk localized prostate cancer [1]. External radiation, such as intensity-modulated radiotherapy (IMRT), stereotactic body radiation therapy, and proton therapy, has been increasingly used in recent years to optimize dose concentration in tumors and reduce exposure to at-risk organs. The 5-year biochemical disease-free survival for external beam radiotherapy was reported to be 80–90% in the low-risk group, 70–80% in the intermediate-risk group, and 50–70% in the high-risk group [2]. Clinical outcomes in the high-risk group can be improved, unlike in the low- to intermediate-risk groups.

Hyperthermia is known to be cytotoxic to cancer cells and acts as a radiosensitizer [3,4]. Radiation therapy-resistant tumor cells that are hypoxic, of low pH, nutritionally deprived, and in the S-phase are more sensitive to hyperthermia [3,5,6]. The clinical efficacy of radiotherapy plus hyperthermia have been demonstrated in randomized clinical trials in patients with advanced head and neck cancer, locally recurrent breast cancer, malignant melanoma, bladder cancer, rectal cancer, and cervical cancer [1]. In patients with prostate cancer, previous phase I/II clinical trials and retrospective studies have described the use of three-dimensional conformal radiation therapy in combination with regional hyperthermia to be both promising and feasible. Additionally, it does not cause severe toxicity [7,8,9,10,11,12,13].

In Japan, the safety and efficacy of hyperthermia in combination with radiotherapy using the 8-MHz capacitive device has been demonstrated since the 1980s, including in prospective phase I/II studies of patients with deep-seated malignant pelvic tumors [14,15,16,17,18]. Based on these results, and since the 1990s, electromagnetic hyperthermia for malignant tumors has been covered by public health insurance, irrespective of the type and stage of the malignant tumor. In Japan, all the people are covered by public health insurance. The patient is free to choose the medical institution and can receive advanced medical treatment at a low cost. In clinical practice, electromagnetic hyperthermia is mainly used in locally advanced cancers wherein further improvement of the antitumor effects of radiotherapy and/or chemotherapy is required, although only a limited number of hospitals are able to carry out the procedure. Hence, in our institution, combination therapy using IMRT and regional hyperthermia was initiated in 2011 to improve the clinical outcomes in patients with high-risk localized prostate cancer. To the best of our knowledge, there are no reports on clinical outcomes after such combination therapy; thus, the purpose of this study was to evaluate the efficacy and toxicity of IMRT plus regional hyperthermia for high-risk localized prostate carcinoma.

## 2. Materials and Methods

### 2.1. Patients

In the current study, we explained to the patients that the standard treatment for National Comprehensive Carcinoma Network (NCCN) high-risk prostate cancer combining IMRT and hormonal therapy results in biochemical recurrence in approximately 20–40% of patients, thereby requiring additional treatment. Furthermore, the possibility of improving the radiotherapeutic effect by performing hyperthermia and the possible side effects (mainly heat sensation, fatigue, and subcutaneous fat burns) were fully clarified. Finally, hyperthermia treatment can only be carried out after the patient had understood the advantages and disadvantages of and consented to the treatment by signing informed consent documents.

This retrospective study was conducted with the permission of the Institutional Review Board of the authors’ university. All personal data, such as names and addresses, were anonymized so that the subjects could not be identified and stored in a locked vault together with their correspondence, under the strict control of the Principal Investigator, when investigating data from electronic medical records and treatment devices.

**Figure 1:**
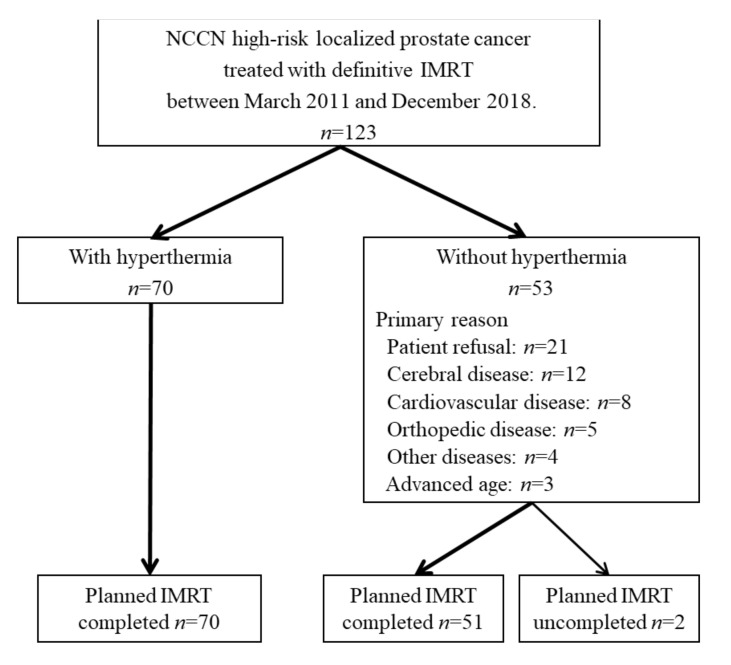
Patient flow diagram.

High-risk prostate carcinoma patients (*n* = 123), defined according to the NCCN, were treated with definitive IMRT between March 2011 and December 2018, at an institutional hospital. During the same period, according to our institution’s treatment protocol aimed at improving clinical outcomes, a subset of the patients (70/123; 57%) were provided regional hyperthermia along with definitive IMRT (Figure 1); the remaining 53 patients were treated with definitive IMRT alone. Primary indications against the use of regional hyperthermia were as follows: patient refusal (*n* = 21), cerebral disease (*n* = 12), cardiovascular disease (*n* = 8), orthopedic disease (*n* = 5), presence of other disease (*n* = 4), and advanced age (*n* = 3). Two of the 123 patients were not able to complete the planned IMRT dose (76 Gy in 38 fractions) and were excluded from the study. Therefore, data from 70 patients treated with definitive IMRT plus regional hyperthermia, and 51 patients treated with definitive IMRT alone, were retrospectively analyzed (Figure 1). Patients with postoperative prostate carcinoma were not included in this study.

Patient baseline characteristics and treatments are listed in Table 1. All patients had pathologically confirmed prostate adenocarcinoma and initially underwent neoadjuvant ADT for a median duration of 9 months (interquartile range, 7–11 months). Adjuvant ADT was continued in 22 patients after completion of IMRT for a median duration of 24 months (interquartile range, 22–33 months). Median total duration of neoadjuvant plus adjuvant ADT was 10 months (interquartile range, 8–18 months).

### 2.2. IMRT

Radiation treatment was provided to all patients with definitive intent using a 10-MV linear accelerator (ONCOR Impression Plus, Siemens Medical Systems, Concord, CA). The clinical target volume (CTV) included the entire prostate, gross extracapsular disease, and proximal seminal vesicles. The planning target volume (PTV) was delineated by contouring the CTV with a margin of 7 mm in all directions except posteriorly, where it was only 4 mm. Our dose prescription policy was based on D95 of the PTV, i.e., percentage of the prescribed dose covering 95% of the volume. The total planned dose for all patients was 76 Gy, with a fractional dose of 2.0 Gy once a day, five times/week. Patients were immobilized using Vac-Lok cushions in the supine position and were treated with step-and-shoot IMRT. A megavoltage cone beam CT system was used to match the patient’s position. Dose-volume constraints for at-risk organs were as follows: rectum V50 Gy < 25%, V65 Gy < 17%; bladder V40 Gy < 50%, V65 Gy < 25%; femoral head D_max_ < 50 Gy, and small intestine D_max_ < 60 Gy.

### 2.3. Hyperthermia

Regional hyperthermia was provided using a 8 MHz radiofrequency capacitive device (Thermotron RF-8, Yamamoto Vinita Co., Osaka, Japan). The physical features of this instrument and its thermal distribution in a phantom model and the human body have been described previously [14,19]. Briefly, both the upper and lower electrodes were 30 cm in diameter and were placed on opposite sides of the pelvis with the patient in the prone position. The treatment goal was at least 30 min of continuous heating after the radiofrequency output was increased to the patient’s tolerance threshold. Patients were carefully instructed to report any unpleasant sensations that were suggestive of a hot spot. Radiofrequency output was increased to the maximum level tolerated by the patient after appropriately adjusting treatment settings. The liquid in the regular boluses adhering to the metal electrode was 5% NaCl or 5% potassium sulfate, both having similar conductivity. To reduce any preferential heating of subcutaneous fat tissue, overlay boluses were applied in addition to regular boluses. Circulating liquid (0.5% NaCl or 0.5% potassium sulfate; both show similar conductivity) inside the overlay boluses was cooled by the RF-8 circulatory system during heating. Superficial cooling was performed using circulating liquid set at 5 °C in the overlay boluses. A gauze soaked in 10% NaCl was inserted in the intergluteal cleft to improve temperature distribution in the prostate. Exceptions occurred in 4 patients provided hyperthermia in 2012; they were included in a previous prospective clinical trial on optimization of deep heating area using this heating device and mobile insulator sheets [20].

Hyperthermia was provided once or twice a week, after radiotherapy. We directly measured intra-rectal temperature in all patients and during all hyperthermia sessions using a 4-point microthermocouple sensor that was inserted into the rectum at the level of the prostate. The thermal dose corresponding to the cumulative equivalent minutes at 43 °C for the T90 (CEM43T90) was obtained based on these intra-rectal temperatures during all hyperthermia sessions. The T90 is an index temperature that indicates either achieving or surpassing 90% of intra-rectal measurement points; similarly, T25 indicates either achievement of target temperature or that it has exceeded 25% of intra-rectal measurement points. The CEM43T90 has been extensively and successfully used in clinical trials to assess efficacy of heating [21,22,23] and provides data on the thermal isoeffect dose expressed in cumulative equivalent minutes at a reference temperature of 43 °C based on the lower end of temperature distribution (T90). The CEM43T90 is calculated from the time-temperature data as follows:

$$\mathrm{CEM}43T90=\sum_{i=0}^{n}t_{i}R^{\left(43-T90i\right)}$$

When the temperature is higher than 43 °C, R = 0.5. When the temperature is lower than 43 °C, R = 0.25. In this protocol, *t_i_* is the time interval of the *ith* sample (*t_i_* = 1.0 min). Temperatures exceeding T90 of the intra-rectal measurement points during the *ith* minute was designated as T90i. We then used the CEM43T90 to convert each T90i into an equivalent time at 43 °C, and these were added over the entire treatment duration of “n” min.

### 2.4. Follow-Up

The length of follow-up was calculated from the IMRT start date. Patients were followed up at intervals of 1–3 months during the first year and at 3–6 months thereafter. At each follow-up visit, PSA was measured, and potential gastrointestinal (GI) and genitourinary (GU) morbidity were accessed. Biochemical relapse was defined as per the Phoenix definition [24]. The presence of bone metastasis was confirmed by bone scintigraphy, CT, or MRI, while soft tissue metastasis was confirmed by CT or MRI. Toxicity of the therapy was evaluated according to the Common Terminology Criteria for Adverse Events, version 4.0. The highest toxicity level for each patient during and after IMRT was used for toxicity analysis. Toxicity was classified as either acute (occurring during therapy or up to 3 months after therapy) or delayed (occurring more than 3 months after completion of therapy).

### 2.5. Statistical Analyses

The Chi-squared test or the Mann–Whitney U test was used to evaluate differences in clinical characteristics between patients with and without hyperthermia. Biochemical disease-free survival (bDFS) (Phoenix definition), clinical relapse-free survival (RFS), and overall survival (OS) rates were calculated from IMRT initiation using the Kaplan–Meier method. Any significant differences between the actuarial curves were assessed using the log-rank test. Hazard ratio and 95% confidence interval were calculated using the Wald test. Multivariate analyses using a Cox proportional hazards model were also performed to identify prognostic factors for the survivals. The Fisher’s exact probability test was used to compare grade 2 or higher toxicity between patients with and without hyperthermia.

## 3. Results

### 3.1. Thermal Data

**Figure 2:**
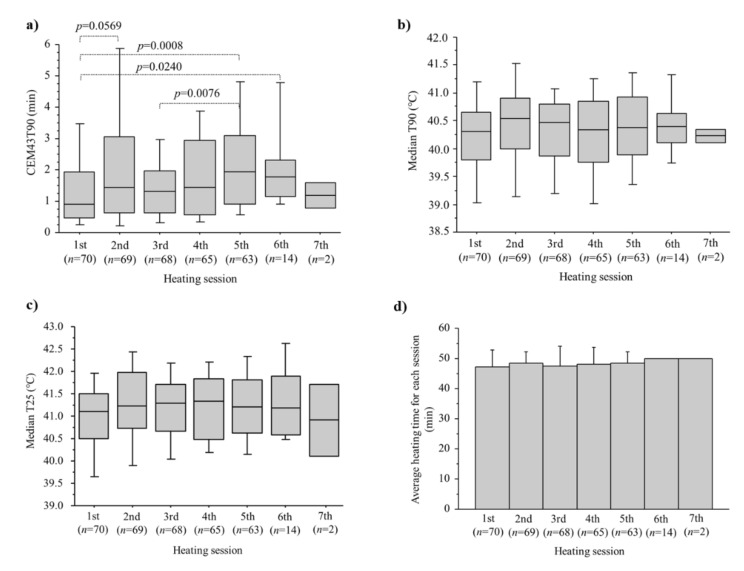
Thermal dose of CEM43T90 (**a**) median T90 (**b**) median T25 (**c**) and heating time (**d**) in each of the HT treatment sessions.

The number of heating sessions in each patient ranged from 1–7 (median, 5) and the median duration of heating per session was 50 min (range, 30–55 min). The thermal dose of CEM43T90 ranged from 0.1 to 32.1 min (median 7.5 min). Figure 2a shows CEM43T90 for each heating session with median values for the first, 2nd, 3rd, 4th, 5th, 6th, and 7th sessions being 0.9, 1.4, 1.3, 1.4, 1.9, 1.8, and 1.2 min, respectively. The CEM43T90 of the first session tended to be lower than of later sessions. Median T90 values for sessions 1–7 were 40.3, 40.5, 40.5, 40.3, 40.4, 40.4, and 40.2 °C, respectively, (Figure 2b) while those for T25 were 41.1, 41.2, 41.3, 41.3, 41.2, 41.2, and 40.9 °C, respectively (Figure 2c). Average heating time for each session is shown in Figure 2d.

### 3.2. Efficacy and Prognostic Factors

Median follow-up time was 64 months (interquartile range, 49–83 months). Table 1 provides data on differences in patient characteristics between the two groups, and no significant differences were detected.

**Figure 3:**
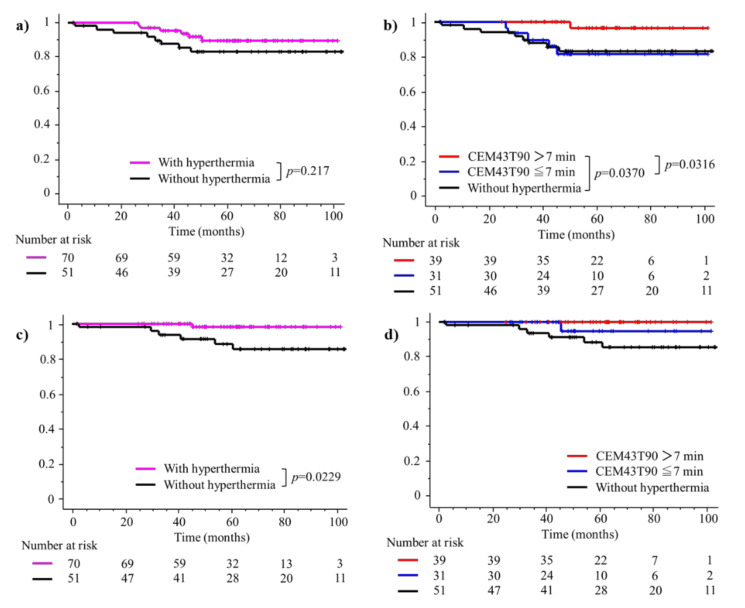
bDFS and clinical RFS rates. (**a**) bDFS with and without hyperthermia treatment. (**b**) bDFS among patients administered a thermal dose of CEM43T90 > 7 min, CEM43T90 ≤ 7 min, or no hyperthermia treatment. (**c**) Comparison of clinical RFS between the groups with and without hyperthermia treatment. (**d**) Comparison of clinical RFS among the patients with thermal dose CEM43T90 > 7 min, CEM43T90 ≤ 7 min, and no hyperthermia treatment.

The 3-year and 5-year bDFS rates were 92.2% and 86.9%, respectively, for all 121 patients and biochemical relapse occurred in 6 patients in each group. Table 2 shows the results of univariate analyses of select factors affecting bDFS, and hyperthermia was not significant predictor of bDFS. Further, 5-year bDFS rate for patients with and without hyperthermia was similar at 89.8% and 82.9%, respectively (*p* = 0.2170, Figure 3a). However, the 5-year bDFS rate was 96.4% in the 39 patients with a CEM43T90 > 7 min, which was significantly better than 82.4% in the remaining 82 patients with a CEM43T90 ≤ 7 min or no hyperthermia treatment (Table 2). Table 3 lists the results of univariate analyses of factors affecting bDFS in 70 patients treated with IMRT plus regional hyperthermia, and a higher thermal dose of CEM43T90 > 7 min was a significant predictor of bDFS. Figure 3b shows that the 5-year bDFS rate of 96.4% in 39 patients with CEM43T90 > 7 min was significantly better than 81.5% in 31 patients with the CEM43T90 ≤ 7 min (*p* = 0.0316) and 82.9% in 51 patients not provided hyperthermia (*p* = 0.0370).

Clinical relapse occurred in one patient treated with hyperthermia and in 4 patients without hyperthermia, and the sites of first clinical relapse were lymph node (*n* = 2), lymph node and lung (*n* = 2), and bone and lymph node (*n* = 1). The 3-year and 5-year clinical RFS rates were 97.4% and 93.9%, respectively, for all 121 patients. Table 4 shows the results of univariate and multivariate analyses of factors related to clinical RFS and additional hyperthermia was significant predictor of clinical RFS in both univariate and multivariate analyses. The 5-year clinical RFS rate was 98.0% for patients provided hyperthermia but 88.6% among patients without hyperthermia (*p* = 0.0229, Figure 3c). Further, 5-year clinical RFS rate was 100% in the 39 patients with CEM43T90 > 7 min and 95.0% in 31 patients with CEM43T90 ≤ 7 min (Figure 3d). The 5-year OS rate was 100% for patients who underwent hyperthermia and 95.9% among patients who did not undergo hyperthermia.

### 3.3. Toxicity

Acute toxicity (≥Grade 2) occurred in 70 patients treated with IMRT and hyperthermia and included grade 2 (*n* = 11, 15.7%) and grade 3 (*n* = 2; 2.8%) GU toxicity. In 51 patients treated with IMRT alone, acute toxicities were grade 3 GU toxicity in 3 (5.9%) patients and grade 2 GU toxicity in 6 (11.8%). The occurrence of acute toxicities ≥ grade 2 was not significantly different between patients with or without hyperthermia treatment. Skin burn, as a subcutaneous induration, was seen in two (2.9%) patients and it spontaneously disappeared after completion of combined therapy. Delayed toxicity ≥ grade 2 among 70 patients treated with IMRT with hyperthermia included grade 3 GI toxicity in one (1.4%) patient and grade 3 GU in one (1.4%) patient. Among 51 patients treated with IMRT alone, delayed toxicity ≥ grade 2 did not occur. Between patients with or without hyperthermia, the occurrence of delayed toxicity ≥ grade 2 was not significantly different.

## 4. Discussion

The results of the present study demonstrate the feasibility of combining IMRT (total 76 Gy in 38 fractions) and regional hyperthermia. This strategy appears to have promising efficacy in patients with high-risk localized prostate carcinoma as the addition of hyperthermia resulted in a significant improvement in clinical RFS. The strengths of this study are that total dose and fractionation of IMRT were identical in all patients, and that neoadjuvant hormone therapy was administered to all patients. Thus, this cohort of patients was suitable for evaluating the radio-sensitizing effect of hyperthermia and for reducing bias due to differences in treatment protocols for NCCN-defined high-risk localized prostate carcinoma. Additionally, temperature in the rectum of the dorsal prostate during heating was monitored in all patients, which permitted adequate analyses of the thermal dose provided.

IMRT is the standard radiation modality used in the treatment of high-risk localized prostate cancer. A recent study with IMRT at a dose of 76–80 Gy plus ADT, which was administrated in 78.5% of the patients with NCCN high-risk localized prostate carcinoma, reported 5-year bDFS and metastasis-free survival rates of 80.6% and 92.5%, respectively [25]. Simizu et al. (2017) have described clinical outcomes after IMRT (72.6–74.8 Gy in 2.2 Gy per fraction) plus ADT administrated to 61% of the patients with high-risk prostate carcinoma and report 5-year bDFS and clinical RFS rates of 77% and 87%, respectively [26]. Marvaso et al. (2018) conducted ultra-hypofractionated radiotherapy using image-guided IMRT (32.5 or 35 Gy in 5 fractions) plus ADT in 21 (75%) of the 28 patients with NCCN high-risk localized prostate carcinoma and report 3-year bDFS and clinical RFS rates of 66% and 87%, respectively [27]. We report higher and more promising 5-year bDFS and clinical RFS rates of 89.8% and 98.0%, respectively, after IMRT with 76Gy in 38 fractions plus regional hyperthermia and ADT (Figure 3a,c).

Previous reports of high-dose IMRT describe the occurrence of acute ≥ grade 2 toxicities to be 28% and that of delayed ≥ grade 2 GI and GU toxicities to be 4% and 15%, respectively, in 772 patients with prostate carcinoma [28]. We have previously reported that addition of regional hyperthermia to 3D-CRT (70 Gy in 35 fractions) did not increase the occurrence of acute or delayed toxicity in patients with prostate carcinoma [13]. Similarly, we now show that acute and delayed toxicities were comparable when regional hyperthermia was added to IMRT.

Maluta et al. (2007) have reported on the clinical outcomes of a prospective phase II study for locally advanced prostate carcinoma in a cohort of 144 patients treated with three-dimensional radiotherapy (74 Gy in 37 fractions) plus regional hyperthermia; additional ADT was administered to more than 60% of the patients [11]. In that study, 5-year OS was 87%, and 5-year bDFS was 49% and no severe toxicities were recorded. Hurwitz et al. (2011) also describe the results of a prospective phase II study for locally advanced prostate carcinoma in 37 patients treated with three-dimensional radiotherapy (66 Gy, daily dose of 1.8–2.0 Gy) plus two transrectal ultrasound hyperthermia treatments and ADT [12,29]; specifically, 5-year OS and bDFS were 93.5% and 60.6%, respectively. Although we only included patients with NCCN high-risk and not very high-risk, IMRT with 76 Gy in 38 fractions plus regional hyperthermia and ADT demonstrated a favorable clinical outcome, indicating that our treatment strategy is promising.

Several clinical randomized trials conducted in the 1990s have demonstrated that adding hyperthermia to radiotherapy improves local control and complete response rates in patients with superficial tumors, such as those involving recurrent breast carcinoma and malignant melanoma [30,31]. Importantly, detailed analyses of thermal data from those randomized trials of breast carcinoma as well as malignant melanoma treated with radiotherapy, with or without hyperthermia, showed significant improvements in local control rates in patients who achieved higher intra-tumor temperatures [32,33]. Previous clinical studies on deep-seated tumors, including cervical carcinoma of the uterus and rectal carcinoma that were treated with hyperthermia plus deep regional hyperthermia, also state that thermal parameters correlate with clinical outcomes [34,35,36]. For prostate carcinoma, we have previously demonstrated that the addition of regional hyperthermia with a higher thermal dose (CEM43T90 ≥ 1 min/heating session) for 3D-conformal radiotherapy improves bDFS [13]. Here, bDFS was significantly higher in patients treated with a higher combined thermal dose of CEM43T90 ≥ 7 min (Figure 3b).

Recent investigations on hyperthermia treatment planning have aimed to simulate temperature patterns as well as specific absorption rate (SAR) distributions, while helping operators visualize the effects of different steering strategies in modern locoregional radiofrequency hyperthermia treatments [37,38,39]. We have previously investigated the use of electromagnetic field numerical simulations for reducing subcutaneous fat overheating, which is a major drawback of deep heating using a capacitively coupled heating system [40]. Hence, optimization of temperature distribution in the deep regional hyperthermia in the pelvis is needed [40] and we used recommended optimal settings in the numerical simulation study, such as use of overlay boluses, electrical conductivity of the circulating coolant, prone position during hyperthermia, and intergluteal cleft gauze, which resulted in improved bDFS among patients who received a good thermal dose. Further improvements in heating methods and selection of patients suitable for hyperthermia represent future research directions.

The efficacy of brachytherapy combined with external beam radiotherapy and ADT as another method of improving the therapeutic effect of IMRT and ADT has been reported in prostate cancer. The ASCENDE-RT trial found that additional low-dose rate brachytherapy improved bDFS, but at the cost of higher, acute and late genitourinary toxicity [41]. Our proposed combination therapy with hyperthermia seems to be a promising method of improving the efficacy of external beam radiotherapy, given its noninvasiveness and the lack of a significant increase in side effects.

Despite these promising results, our study has a few limitations. As this was a retrospective study, the possibility of selection bias with respect to prognostic factors cannot be ruled out. However, as dose prescription for IMRT was constant and there were no differences in the major prognostic factors between patients with and without hyperthermia, the influence of selection bias can be presumed to be relatively small. The duration of ADT was a potential confounding factor. Although no significant difference was found in the duration of ADT between the patients with and without hyperthermia treatment, the duration of ADT was shorter in the hyperthermia group. Therefore, we speculate that the duration of ADT is unlikely to be a confounding factor in the results of this study. A formal prospective clinical trial is needed to determine the efficacy and prognostic factors associated with this approach of combined therapy in patients with high-risk localized prostate carcinoma.

## 5. Conclusions

To the best of our knowledge, this is the first report to assess efficacy, in terms of clinical outcomes, of a combination of IMRT and regional hyperthermia in patients with high-risk localized prostate carcinoma. We demonstrate that the use of definitive IMRT, combined with regional hyperthermia, is a promising treatment modality that is not associated with severe toxicity. Our results support further evaluation such as clinical trials evaluating IMRT with or without regional hyperthermia in patients with high-risk localized prostate carcinoma.

## Figures and Tables

**Table 1:** Patient characteristics.

Characteristics	With Hyperthermia	Without Hyperthermia	*p*
	*n* = 70 (%)	*n* = 51 (%)	
Age (median, range)	72 (54–80)	71 (54–83)	0.3381
Performance status			0.1948
0	41 (59)	25 (49)	
1	29 (41)	23 (45)	
2	0	2 (4)	
3	0	1 (2)	
T stage			0.8000
T1	25 (36)	18 (35)	
T2	31 (44)	25 (49)	
T3a	14 (20)	8 (16)	
N stage			
N0	72 (100)	51 (100)	
Gleason score			0.4774
≤7	17 (24)	14 (28)	
8	25 (36)	22 (43)	
9–10	28 (40)	15 (29)	
Pretreatment PSA (ng/mL)			0.6095
<10	20 (29)	17 (33)	
10–20	19 (27)	16 (31)	
>20	31 (44)	18 (35)	
IMRT			
76 Gy, 38 fractions	72 (100)	51 (100)	
Total ADT duration			0.2296
<6 months	2 (3)	0 (0)	
6–11 months	46 (66)	29 (57)	
≥12 months	22 (31)	22 (43)	
Hyperthermia			
Number of sessions			
1	1 (1)	-	
2	1 (1)	-	
3	3 (4)	-	
4	2 (3)	-	
5	49 (70)	-	
6	12 (17)	-	
7	2 (3)	-	

PSA, prostate-specific antigen; IMRT, intensity-modulated radiotherapy; ADT, androgen deprivation therapy.

**Table 2:** Univariate analyses of certain factors for bDFS in 121 patients treated with IMRT with or without regional hyperthermia.

Variation	Patients (*n*)	5-y (%)	*p* (Log-Rank Test)	Hazard Ratio * (95% Confidence Interval)
T stage				
T1–T2	99	87.4	0.6978	0.777 (0.217–2.786)
T3a	22	84.0		
Gleason score				
≤8	78	86.8	0.8710	0.913 (0.306–2.726)
≥9	43	87.2		
Pretreatment PSA (ng/mL)				
≤20	72	88.4	0.4478	0.668 (0.234–1.905)
>20	49	84.8		
Total ADT (months)				
≤10	70	84.0	0.3344	0.569 (0.178–1.815)
>10	51	91.3		
Hyperthermia				
Yes	70	89.8	0.2170	0.519 (0.180–1.497)
None	51	82.9		
Hyperthermia				
CEM43T90 > 7	39	96.4	0.0296	0.144 (0.019–1.099)
None or CEM43T90 ≤ 7	82	82.4		

* Hazard ratio and 95% confidence interval were calculated using the Wald test.

**Table 3:** Univariate analyses of certain factors for bDFS in 70 patients treated with IMRT plus regional hyperthermia.

Variation	Patients (*n*)	5-y (%)	*p* (Log-Rank Test)	Hazard Ratio * (95% Confidence Interval)
T stage				
T1–T2	56	89.0	0.8403	0.802 (0.094–6.869)
T3a	14	92.9		
Gleason score				
≤8	42	91.2	0.5298	0.602 (0.121–2.984)
≥9	28	87.7		
Pretreatment PSA (ng/mL)				
≤20	39	89.7	0.784	0.800 (0.161–3.964)
>20	31	89.7		
Total ADT (months)				
≤10	42	86.3	0.2986	0.338 (0.039–2.894)
>10	28	96.3		
Hyperthermia				
CEM43T90 (min)				
≤7	31	81.5	0.0316	0.134 (0.016–1.152)
>7	39	96.4		

* Hazard ratio and 95% confidence interval were calculated using the Wald test.

**Table 4:** Univariate and multivariate analyses of certain factors for clinical relapse-free survival in 121 patients treated with IMRT with or without regional hyperthermia.

Variation	Patients (*n*)	Univariate			Multivariate	
		5-y (%)	*p* *	Hazard Ratio ** (95% CI)	*p*	Hazard Ratio (95% CI)
T stage						
T1–T2	99	94.2	0.5391	0.601 (0.116–3.107)	0.564	0.600 (0.106–3.403)
T3a	22	93.3				
Gleason score						
≤8	78	95.7	0.5723	0.651 (0.145–2.920)	0.317	0.455 (0.097–2.125)
≥9	43	90.3				
Pretreatment PSA (ng/mL)						
≤20	72	92.8	0.5504	0.610 (0.118–3.144)	0.597	0.612 (0.100–3.766)
>20	49	95.5				
Total ADT (months)						
≤10	70	91.5	0.1592	0.246 (0.030–2.043)	0.121	0.170 (0.018–1.599)
>10	51	97.4				
Hyperthermia						
Yes	70	98.0	0.0229	0.126 (0.015–1.049)	0.035	0.099 (0.000–0.852)
None	51	88.6				

* Log-rank test. ** Hazard ratio and 95% confidence interval were calculated using the Wald test. CI, confidence interval; PSA, prostate-specific antigen; ADT, androgen deprivation therapy.

## Data Availability

The data presented in this study are available on request from the corresponding author.
